# A perceptive plus in Parkinson's disease

**DOI:** 10.1002/mds.27240

**Published:** 2018-01-14

**Authors:** Marc M. Himmelberg, Ryan J.H. West, Alex R. Wade, Christopher J.H. Elliott

**Affiliations:** ^1^ Department of Psychology The University of York York UK; ^2^ Department of Biology The University of York York UK

Beard JD , Steege AL , Ju J , Lu J , Luckhaupt SE , Schubauer‐Berigan MK . Mortality from amyotrophic lateral sclerosis and Parkinson's disease among different occupation groups—United States, 1985–2011. MMWR Morb Mortal Wkly Rep 2017;66(27):718‐722. 2870434610.15585/mmwr.mm6627a2PMC5687590

The puzzle of Parkinson's disease (PD) is particularly elusive, but the next part of the picture is appearing, and it is a curious one: a tale of men, mice, and flies. Recently, Beard and colleagues^1^ reported that people who went on to develop PD tended to have jobs with higher socioeconomic status. Their study of > 12 million Americans highlighted more than 110,000 deaths from PD, with excess numbers of workers in community services (48%), educational (46%), legal (40%) and the sciences (33%). Such jobs may be demanding of deeper thought, good discrimination, and quick judgments. In a second study of >4.5 million people from the Swedish census, those with lower socioeconomic status had a lower PD incidence.^2^

Although this may appear (at first sight) far‐fetched, advantages in cognition in people at risk of PD are predicted from our studies of young PD‐mimic flies. These have faster, stronger visual responses^3^, ^4^ when the flies are young; however, in old age they show a loss of response and neurodegeneration. This model is noteworthy because ever since the time of Cajal, the homology of vertebrate and fly visual systems has been recognized, with many similarities at the neural circuit, computational, and developmental levels. Crucially, both flies and vertebrates use dopamine for retinal gain control. Furthermore, it is widely accepted that the extra demand for energy is a major cause of neurodegeneration in PD, so that the loss of visual gain control in young flies will lead to increased visual responses, requiring more Adenosine Triphosphate to pump ions and maintain synaptic transmission.

Increased visual processing, and possibly faster neural signaling, as a result of deficits in retinal dopamine signaling may provide people at risk of PD with advantages in younger life, which impact before the later neurodegeneration. They may be more suited to jobs with higher socioeconomic status, both at interview and in the daily routine. This would explain the new observations.^1^, ^2^ Furthermore, PD‐linked mutations have been around since prehistoric times^5^ and may therefore have had a selective advantage for young people encountering situations demanding rapid responses, for example, escape or hunting activities.


Marc M. Himmelberg, BA, BPsych^1^ Ryan J.H. West, BSc, PhD^2^ Alex R. Wade, BA, PhD^1^ Christopher J.H. Elliott, MA, DPhil^2^
^1^*Department of Psychology, The University of York, York, UK*
^2^*Department of Biology, The University of York, York, UK*

## Author Roles

1) Research project: A. Conception, B. Organization, C. Execution; 2) Statistical Analysis: A. Design, B. Execution, C. Review and Critique; 3) Manuscript: A. Writing of the first draft, B. Review and Critique.

M.H.M.: 3B

R.J.H.W.: 3B

A.R.W.: 3B

C.J.H.E.: 3A

## Financial disclosures of all authors (for the preceding 12 months)

MMH was supported by the European Union's Horizon 2020 research and innovation programme under the Marie Sklodowska‐Curie grant agreement No 641805. R.J.H.W. have nothing to disclose. A.R.W. and C.J.H.E. have (in the past 12 months) grants and nonfinancial support from Lundbeck A/S outside the submitted work. A.R.W. has support from Biotechnology and Biological Sciences Research Council (BBSRC).
